# Molecular Chaperone HSPA2 Distribution During Hyaluronic Acid Selection in Human Sperm

**DOI:** 10.1007/s43032-022-01031-9

**Published:** 2022-07-11

**Authors:** María José Gómez-Torres, Natalia Huerta-Retamal, Paula Sáez-Espinosa, Laura Robles-Gómez, Manuel Avilés, Jon Aizpurua

**Affiliations:** 1 Departamento de Biotecnología, Universidad de Alicante, 03690 Alicante, Spain; 2 Departamento de Biología Celular E Histología, Universidad de Murcia, Instituto Murciano de Investigación Sociosanitaria (IMIB-Arrixaca), 30003 Murcia, Spain; 3 IVF Spain, Medicina Reproductiva, 03540 Alicante, Spain; 4 Cátedra Human Fertility, Universidad de Alicante, Alicante, Spain

**Keywords:** Heat shock protein, HSP70, Immunofluorescence, Male infertility, Sperm selection

## Abstract

During fertilization, sperm hyaluronidase activity is essential for spermatozoa to successfully penetrate the hyaluronic acid-enriched extracellular matrix of the cumulus cells. Since molecular chaperones, as the heat shock protein A2, are typically involved in bringing hyaluronic acid receptors to the cell surface, here we evaluated the presence and spatial location of HSPA2 on human spermatozoa based on its hyaluronic acid binding capacity. This study included 16 normozoospermic sperm samples from volunteering donors. The location of HSPA2 was studied in cells before and after 1-h incubation under capacitating conditions, as well as in spermatozoa selected according to their ability of binding to hyaluronic acid. Our results showed no significant differences in HSPA2 immunofluorescent cells before and after 1 h of incubation in capacitating conditions. Nevertheless, after hyaluronic acid selection, the percentage of HSPA2-labelled cells increased significantly, indicating that the interaction with hyaluronic acid may induce the unmasking of HSPA2 epitopes. Furthermore, after swim-up and hyaluronic acid selection, spermatozoa presented a highly immunostained equatorial band with a homogeneous fluorescence throughout the acrosomal region. This distribution has been previously suggested to have important implications in male fertility. Noteworthy, a homogeneous fluorescence among the acrosomal region with a more intense labelling at the apical region was observed only in hyaluronic acid bound sperm cells, which may be associated with primary gamete recognition. Our findings suggest that the hyaluronic acid selection technique and HSPA2 biomarker should be considered candidates to complement the classic seminal analysis before recommending an appropriate assisted reproduction technique.

## Introduction

The ability of sperm to fertilize the oocyte is acquired in vivo during their transit through the female tract. This process, named “capacitation,” is vital for the sperm to effectively interact with the female gamete [1–5]. During capacitation, the female tract environment causes a number of physiological changes in the sperm, such as a loss of cholesterol [3], reorganization of glycoconjugates [6], phosphorylation of proteins [7], and hyperactivation [8]. Certainly, previous studies have demonstrated the physiological importance of tyrosine phosphorylation in fertilization [9], pointing to flagellar tyrosine phosphorylation as a reliable biomarker for capacitation [10, 11].

The possibility to perform in vitro capacitation, among other innovations, has enabled the development of an effective treatment for male reproductive dysfunction using intracytoplasmic sperm injection (ICSI) [12, 13]. ICSI is currently the most widely used technique for fertilization in assisted reproduction clinics due to its benefits in patients with low seminal quality [14–16]. However, the ICSI success rate remains with a full-term pregnancy cycle of approximately 24% for all age groups [15, 17]. This reduced success rate may be related to the fact that this procedure overcomes all the physiological barriers involved in the selection of an appropriate spermatozoon [17–21]. Moreover, the current sperm selection technique is performed visually focusing on sperm morphology and motility. Overall, the current methodology affects embryo development, in addition to increasing the risk of genetic defects [18–23]. Therefore, improvement of the sperm selection technique prior to ICSI is a particularly required issue.

In this context, the test for hyaluronic acid (HA) binding capacity is a useful and non-invasive method of sperm selection. This technique is based on its ability to capture only spermatozoa expressing receptors for HA. This assay has been previously described to select sperm cells more likely to have normal morphology, low levels of DNA fragmentation, low rates of chromosomal aneuploidy [18, 21, 24, 25], normal cytoplasmic extrusion, and plasma membrane remodeling [18, 24, 26–28]. Moreover, compared to conventional ICSI, a direct increase in fertilization, implantation rates, as well as in subsequent embryo quality and pregnancy, has been observed [26, 27, 29, 30].

Furthermore, HA may perform a significant role in fertilization since, during natural fertilization, only sperm cells able to penetrate through the HA-enriched extracellular matrix surrounding the cumulus cells would fertilize the oocyte [31]. Therefore, sperm hyaluronidase activity is essential for the access and later binding to the zona pellucida (ZP) [21, 24, 32, 33]. In order to penetrate through these cells, HA receptors need to be brought to the cell surface and/or assembled into functional complexes by molecular chaperones [34, 35]. Among the chaperones involved in this process in human spermatozoa, the heat shock protein A2 (HSPA2) has been considered a key focus of study [36]. This chaperone participates in the dynamics of the hyaluronidase sperm adhesion molecule 1 (SPAM1) and the zona pellucida receptor molecule Arylsulfatase A (ARSA) [37]. In addition, the reduced representation of HSPA2 on sperm cells is related to a low number of ZP binding sites [37–39] and hyaluronan receptors on the sperm [24].

The importance of HSPA2 during spermatogenesis and fertilization has been previously established given the infertility phenotype of knockout for this gene in mice. [38, 40]. The most relevant studies in human samples have reported reduced levels of HSPA2 expression in samples from patients suffering varicocele [41, 42], elevated ROS (reactive oxygen species) levels [43], infertility [44], or disability to bind to the ZP [45]. Moreover, a recent report demonstrates the association between lower HSPA2 expression with recurrent pregnancy loss, indicating the potential usefulness of HSPA2 in the diagnosis and prognosis of paternal effects [46].

Furthermore, the proportional relationship between the success of fertilization using ICSI and the presence of testicular HSPA2 [47] has made possible to conclude the value of HSPA2 as a biomarker of success for ICSI and IVF (in vitro fertilization) [47–49]. Otherwise, previous studies have demonstrated a positive relationship among HSPA2 expression and fertility potential of a semen sample in patients with ZP binding deficiency [39, 44, 45] and varicocele [41, 50].

Relevant reports about HSPA2 location are based on the assumption that the protein co-localizes with ARSA and SPAM1 in the acrosomal region of capacitated sperm's head [37, 39]. However, this hypothesis is controversial because other authors have reported that this chaperone is distributed in different regions of the sperm head (acrosome, postacrosomal, and equatorial zone) and these different distributions correlate with fertility [39].

Taken collectively, it can be concluded that the presence of HSPA2, the binding with HA, and the interaction of spermatozoa with the ZP are functionally linked together and are also associated with male infertility. Thus, without HSPA2, the receptor of the hyaluronidase (SPAM1) and the receptor of the ZP (ARSA) would not be expressed in the coordinated manner necessary to achieve fertilization [45]. Therefore, and considering the current low full-term pregnancy rate after ICSI and the HSPA2 importance in sperm maturation, the aim of this study was to know the presence and spatial location of HSPA2 on human sperm according to their HA binding capacity.

## Materials and Methods

### Seminal Sample Analysis

The semen samples used for this study were obtained after an informed written consent from 16 normozoospermic donors later 3 to 4 days of abstinence. Prior to 1 h, a basic semen analysis was performed and only those samples classified as normozoospermic according to World Health Organization were included [51]. The samples were then divided into two aliquots to be studied before (uncapacitated sperm US) and after in vitro incubation under capacitating conditions (capacitated sperm CS). CS were then selected by HA binding assay based on previous study [26]. This research was approved by the Bioethics Committee of the University of Alicante (Spain) in accordance with the principle of the Declaration of Helsinki.

### In Vitro Incubation Under Capacitating Conditions

The seminal plasma was removed by centrifugation for 10 min at 250 g at room temperature; then, a wash with human tubal fluid medium (HTF, Origio®, Måløv, Denmark) for 5 min at 250 g was performed. The sample was then selected by the swim-up technique for 1 h using HTF supplemented with 5 mg/ml of bovine serum albumin (BSA, Sigma-Aldrich®, Saint Louis, Missouri, USA) at 37 °C and 5% (v/v) CO_2_. The incubation time (1 h) was used according to WHO criteria [51].

Following incubation, supernatant fraction was collected and washed in phosphate buffered saline (PBS, Life Biowest, Nuaillé, France). Before and after incubation in capacitating media, samples were fixed in 2% (w/v) paraformaldehyde (Electron Microscopy Sciences, Hatfield, Pennsylvania, USA) for 1 h at 4 °C. Finally, they were re-suspended in PBS to reach a final concentration of 1 × 10^6^ sperm cells/ml and preserved at 4 °C.

### Sperm Selection

**Fig. 1:**
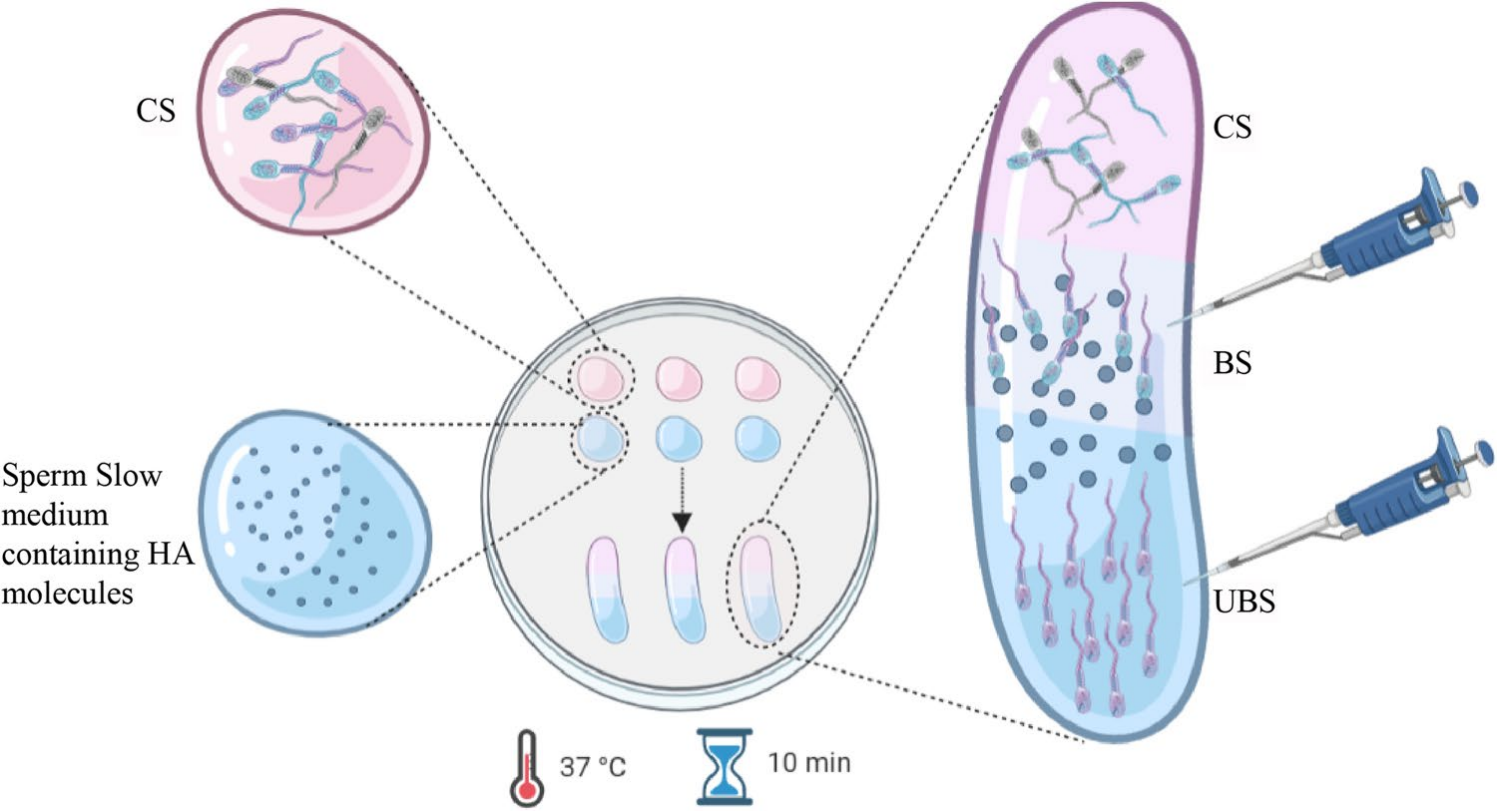
Experimental design. Schematic description of the experimental design followed in this study to select the previously capacitated sperm cells (**CS**) using Sperm-Slow™ medium. After a 10-min incubation at 37 °C, spermatozoa which had been able to bind hyaluronic acid (**HA**) were recovered as **BS**. In the same way, those cells that failed to bind HA were collected and categorized as **UBS**. A total of 250–300 cells were recovered from BS and UBS groups. Created with BioRender.com

A 15-µL droplet from CS was connected with a pipette tip to a 15-µL droplet of Sperm-Slow™ medium (Origio®) containing HA on a plastic culture dish. They were then incubated for 10 min at 37 °C under oil (FertiCult™ Mineral Oil, FertiPro, Beemen, Belgium) following manufacturer’s instructions. Spermatozoa with HA receptors were able to bind the HA, whereas spermatozoa lacking these receptors swam through the Sperm-Slow™ droplet. Spermatozoa bound to HA were slowed in the junction zone of the two droplets; these spermatozoa were collected and subsequently placed into a coverslip named as bound sperm (BS). Not bound spermatozoa was moving freely at the bottom of the HA droplet; these cells were collected and placed into a coverslip named as unbound sperm (UBS). In order to clarify the experimental design, an illustrative figure is included (Fig. 1). The percentage of HA-bound sperm was calculated as following: BS/CS × 100.

Cells of each experimental condition (US, CS, BS, and UBS) were then air-dried and fixed in 2% (v/v) of paraformaldehyde during 1 h at 4 °C and conserved at 4 °C after three washes in PBS.

### Tyrosine Phosphorylation

In order to quantify one of the molecular changes taking place during capacitation, flagellum tyrosine phosphorylation was analysed. The assays were based on standard procedures, as described previously [52]. Fixed cells were permeabilized in 0.1% (v/v) Triton X-100 (Sigma-Aldrich®) for 10 min. After PBS washing, unspecific binding was blocked by incubating with 2% (w/v) BSA-PBS for 30 min. Phosphorylated tyrosine was labelled by incubating with a mouse monoclonal anti-phosphotyrosine antibody (PY20, Sigma-Aldrich® Cat# P4110, RRID: AB_477342) at 1:500 dilution in PBS for 1 h, followed by a polyclonal donkey anti-mouse cyanine-3 antibody (Jackson ImmunoResearch Labs, Ely, UK Cat# 715–165-150, RRID: AB_2340813) at 1:300 dilution in PBS for 1 h in darkness. After a final 15-min wash in PBS, the coverslips were mounted with Vectashield® H-100 mounting medium containing DAPI (Vector Laboratories, Burlingame, California, USA). The assessment of flagellum tyrosine phosphorylation was carried out evaluating about 300 cells in each sample and experimental condition, for a total of 19,200 cells assessed. Anti-phosphotyrosine antibody was omitted for negative control.

### Heat Shock Protein A2 (HSPA2) Immunolocalization

In order to assess the HSPA2 localization on the spermatozoa and according the previously described procedure [53], we adapted previous protocol described by Motiei et al. [36, 39]. Briefly, previously fixed samples were placed on coverslips and, once dried, spermatozoa were rehydrated in PBS for 15 min at 5-min intervals and permeabilized with Triton X-100 at 0.2% (v/v) in PBS for 10 min at room temperature. After permeabilization and in order to prevent unspecified binding sites, the coverslips were incubated with the primary rabbit polyclonal anti-HSPA2 antibody solution in phosphate-buffered saline, pH 7.2, containing 40% glycerol and 0.02% sodium azide (Cat. Number HPA000798, Sigma-Aldrich, Inc.) at a final concentration of 1:100 in blocking solution (PBS-BSA 3% (w/v)) overnight. After being washed with PBS three times for 5 min, samples were incubated for 1 h in the dark with a polyclonal donkey anti-rabbit IgG-FITC (Thermo Fisher Scientific Cat# 31,568, RRID: AB_228234) antibody diluted 1:100 in blocking solution. Finally, three washes in PBS were carried out at 5-min intervals. After dry, the samples were mounted using Vectashield® H-100 mounting medium which contains DAPI (Vector Laboratories). The evaluation of staining patterns was carried out by assessing approximately 300 cells in each sample and experimental condition, making a total of 19,200 cells evaluated. Primary antibody was omitted for negative controls. The quantification of staining patterns was carried out using the Zeiss LSM 800 confocal laser microscope (Zeiss, Oberkochen, Germany) and Zeiss Confocal Software.

### Statistical Analysis

Owing to the normal distribution (Shapiro–Wilk test; *p* > 0.05), statistical differences were analysed using two-way analysis of variance (ANOVA) followed by univariate analysis and Bonferroni post hoc tests. When analysing HSPA2 distribution, only the patterns present in more than 5% of sperm in a sample, regardless of the experimental condition, were considered. Descriptive and statistical results were obtained using IBM SPSS Statistics 22.0 (IBM, Armonk, NY, USA, RRID:SCR_002865). Two-sided *p*-values < 0.05 were considered to be statistically significant.

## Results

### Seminal Sample Analysis

A cohort of healthy male volunteers (*n* = 16) was used in this study. According to WHO reference values [51], all included samples were categorized as normozoospermic. The mean and standard deviation of sperm concentration, the percentage of sperm motility, normal morphology, in each experimental condition and the average sperm bound to HA are showed in Table 1.

**Table 1:** Seminal analysis result for samples used in the study (mean (%) ± standard deviation) before (US) and after incubation under capacitating conditions (CS). Bonferroni post hoc test, significant differences *p* < 0.01 (*)

Parameter	Mean ± *σ*	
	US	CS
Sperm concentration (× 10^6^ cells/ml)	68.2 ± 22.3	5.7 ± 2.8*
Progressive motility (%)	70.2 ± 14.1	96.1 ± 4.6*
Normal morphology (%)	13.1 ± 6.5	18.8 ± 5.9*
HA-bound sperm (%)	-	21.4 ± 7.4

### Tyrosine Phosphorylation

**Fig. 2:**
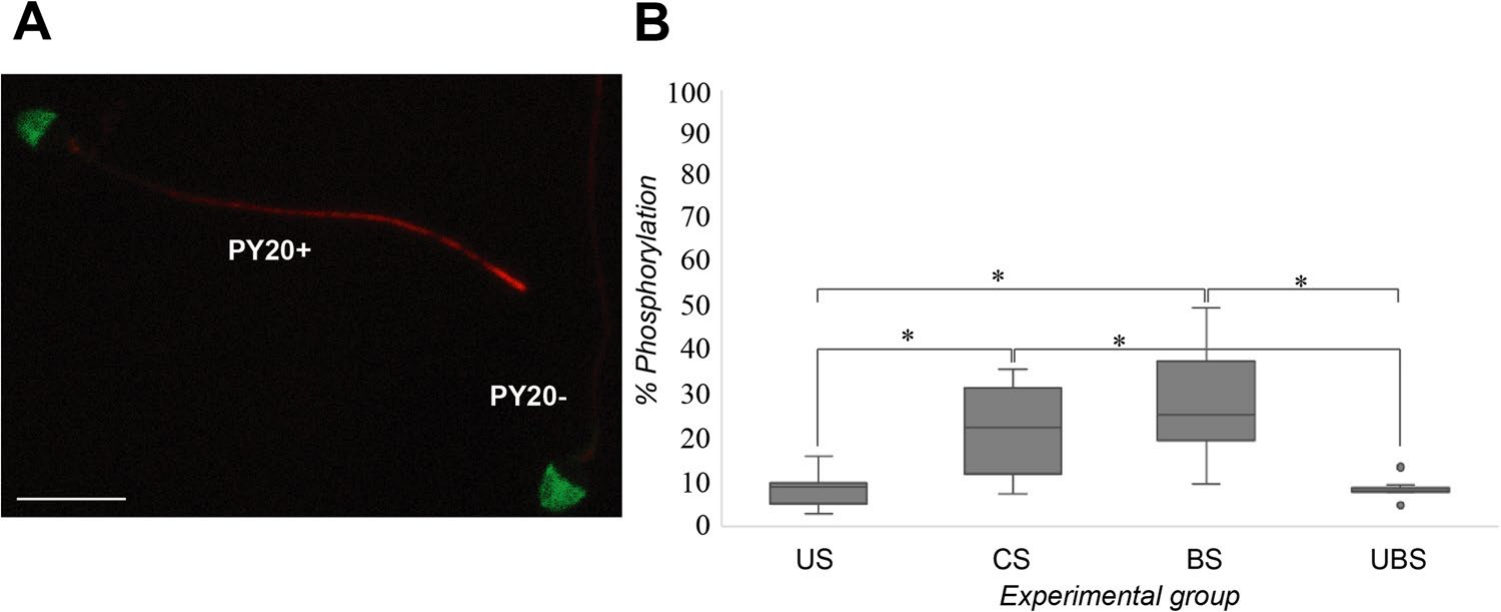
Tyrosine phosphorylation results. **A** Fluorescence patterns found in the analysis of tyrosine phosphorylation. Sperm cells showing partial or complete fluorescence in their flagellum were classified as phosphorylated (**PY20 +**) while those without observed fluorescence were recorded as **PY20** − . Scale bar: 10 µm. **B** Tyrosine phosphorylation results expressed as mean + / − 95% interval confidence (*n* = 16) in each condition used in the present study. Uncapacitated sperm (**US**), capacitated sperm (**CS**), hyaluronic acid-bound sperm (**BS**), and unbound sperm after hyaluronic acid-assay (**UBS**). Bonferroni post hoc test, significant differences in *p* < 0.05 (*)

Spermatozoa were classified as phosphorylated when they had partial or complete fluorescence in their flagellum [52, 54] (Fig. 2A). A significant increase in sperm with phosphorylated tyrosine after incubation under capacitating conditions was observed. In US, 8.5% of the cells showed phosphorylated tyrosine in their flagellum. After swim-up, this percentage significantly increased (Bonferroni difference (BD); *p* = 0.007) to 24.7%. Moreover, once the spermatozoa were HA selected, 26.4% of sperm was phosphorylated in BS (significant difference with US sperm; BD: *p* < 0.001); meanwhile, only 8.1% of cells presented a partial or complete fluorescence in their flagellum in UBS, with significant differences with CS and BS (BD; *p* < 0.05) (Fig. 2B).

### Distribution of the Heat Shock Protein A2 (HSPA2)

**Fig. 3:**
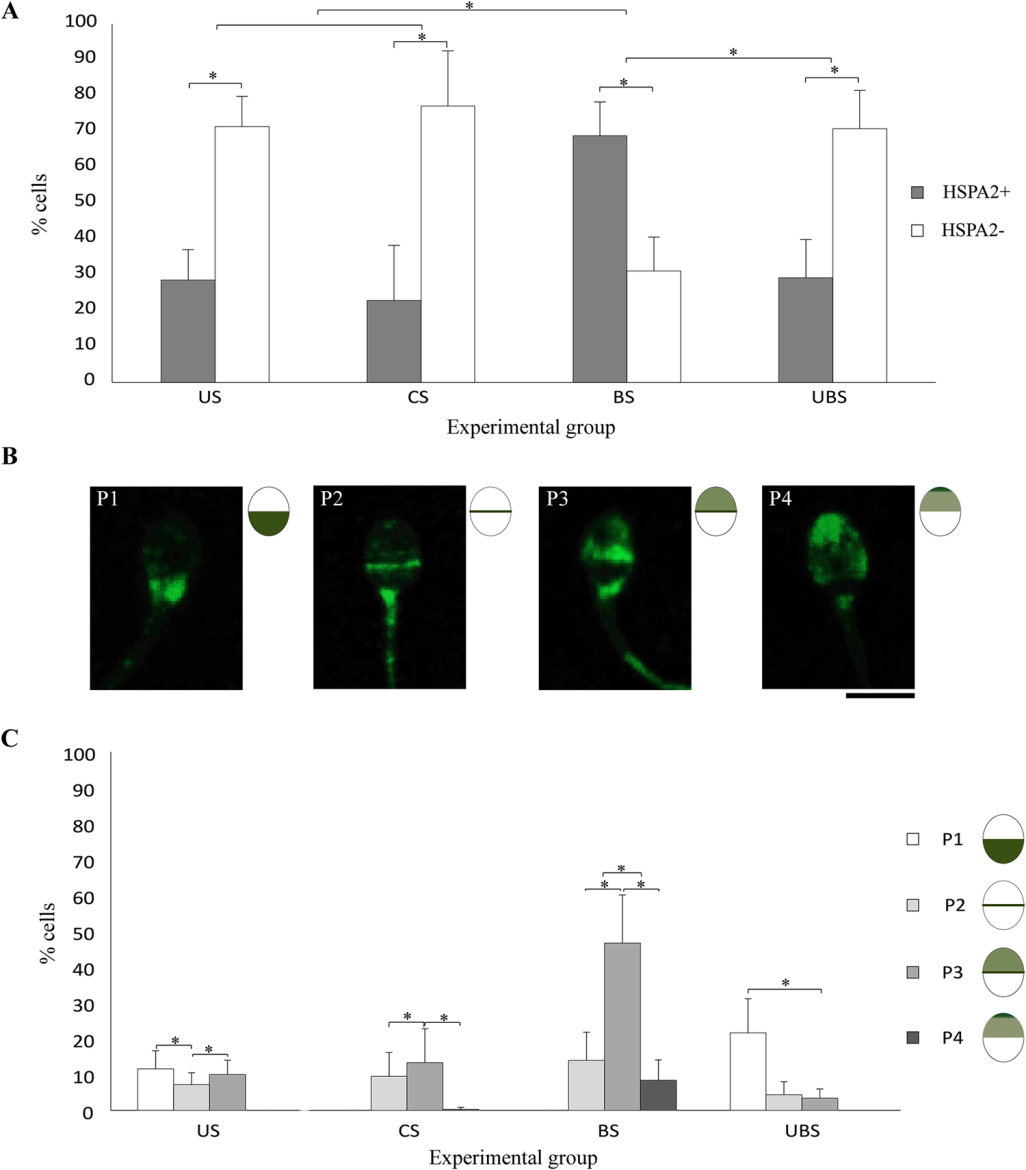
HSPA2 evaluation results. **A** Frequency of cells with and without immunofluorescence for HSPA2 (**HSPA2 + , HSPA2** −) in each physiological condition (i.e., uncapacitated sperm (**US**), capacitated sperm (**CS**), hyaluronic acid-bound sperm (**BS**), hyaluronic acid-unbound sperm (**UBS**)). **B** Fluorescence patterns observed: labelling in the postacrosomal region (**P1**), fluorescence in the equatorial band (**P2**), intense fluorescence in the equatorial band accompanied by decreased immunolabelling in the acrosomal region (**P3**), homogeneous fluorescence throughout the acrosomal region with increased apical area (**P4**). 5 µm scale common to all images. **C** Frequency of HSPA2 patterns in human spermatozoa that showed immunofluorescence for HSPA2 (HSPA2 +) in the different experimental groups. Bonferroni post hoc test, significant differences in *p* < 0.05 (*)

The evaluation of the presence of HSPA2 in the sperm head showed that there was no statistical difference between the percentages of labelled cells in US and CS groups (28.6% and 22.9%, respectively). However, in BS, we observed a significant increase of labelled cells for HSPA2 (68.9%, BD; *p* < 0.001). Nevertheless, in UBS, only 29.2% of cells presented fluorescence for HSPA2, showing no statistical differences with US nor CS but significant difference with BS (BD; *p* < 0.001) (Fig. 3A).

Alternatively, the assessment of HSPA2 distribution in labelled sperm led us to differentiate four distribution patterns (Fig. 3B). The pattern 1 (P1) was characterized by an intense fluorescence in the post-acrosomal region; pattern 2 (P2) was expressed in the equatorial band; the pattern 3 (P3) displayed fluorescence in the equatorial region with a less intense and homogeneous labelling throughout the acrosomal region and pattern 4 (P4) showed homogeneous fluorescence among the acrosomal region with a more intense labelling at the top of the acrosome. Not all patterns were observed in all experimental conditions (Fig. 3C).

In US sperm showing fluorescence for HSPA2, the main patterns observed were P1 and P3 (11.5% and 9.9% of cells respectively). Instead, P2 was significantly less represented (BD; *p* < 0.001) with a 7.2% of cells (Fig. 3C). In contrast, in CS sperm, the pattern P1 was not found, and P3 resulted to be the significative most representative pattern, accounting for 13.3% and showing no significant differences with US. Similarly, P2 did not show any significant difference with US, being observed in the 9.4% of CS labelled cells. Furthermore, in this group, a low and not significant number of sperm cells (0.2%) presented P4, a pattern not observed in US (Fig. 3C).

On the other hand, after HA selection, P1 was not found in BS group. However, P2 showed a significant increase (BD; *p* < 0.001) (13.9%) compared US. The most representative pattern in this experimental condition was P3, with 46.6% of representation. This pattern increased significantly (BD; *p* < 0.001) in comparison with US and CS cells. Meanwhile, (P4) was present in 8.3% of the BS cells, increasing significantly compared to CS (BD; *p* < 0.001) (Fig. 3C).

In contrast, the comparison of HSPA2 protein location between BS and UBS showed significant difference (BD; *p* < 0.001) on the representation of every pattern observed. Firstly, the presence of P1 in UBS increased (21.5%) (BD; *p* < 0.001) against BS as well as US and CS groups, to become the predominant distribution pattern. Additionally, P3 decreased to become the less observed pattern in UBS (3.3%; BD; *p* < 0.001 against CS). Finally, as in US cells, P2 was observed in 4.3% of the cells, whereas P4 was not observed (Fig. 3C).

## Discussion

In the last decade, studies regarding sperm selection have focused on strategies to improve the recovery of a viable and fertile sperm population for ICSI and IVF [17, 18, 51]. However, the functional properties of the selected sperm cell, such as DNA integrity, sperm maturity, or tyrosine phosphorylation, are often not considered during routine sperm preparation [51].

Among some promising procedures regarding sperm selection [18, 20, 55–59], HA binding selection could be a reliable method. This technique is based on the capacity of HA to retain spermatozoa expressing proteins that may facilitate the penetration of spermatozoa through the cumulus matrix by hyaluronidase activity [18, 21, 25, 60].

Despite the biological value of HA selection, there is controversy regarding the benefits of this technique over conventional ICSI. While some research shows a positive correlation between hyaluronic acid binding and seminal parameters such as motility and morphology [61], other reports such as Miller et al. in 2019 [62] conclude that there is no significant improvement in the ICSI outcomes by using the HA binding assay. However, a recent paper has demonstrated the usefulness of this assay in older couples to improve fertilisation and live birth rates [63].

Since HSPA2 regulates the location of ARSA and SPAM1 [35–37, 45], the study of HSPA2 dynamics after HA selection may lighten the viability of this protein as a biomarker of maturity, as well as to elucidate the aptitude of HA selection method to sort useful spermatozoa for ICSI. To the best of our knowledge, no studies have mapped the spatial and temporal behaviour of HSPA2 in HA-selected human sperm.

Considering that flagellar tyrosine phosphorylation is one of the molecular events taking place during capacitation and is essential throughout the fertilization process [36], it was assessed after incubation and cell selection. Our results showed an incubation-dependent increase in tail tyrosine phosphorylation consistent with previous studies [10, 36]. Nevertheless, the results of our study go further, as they also showed a significantly higher percentage of phosphorylated cells in the group of cells that were able to bind to HA than in the group of US and UBS. From these results, we can confirm the adequacy of the incubation times used during both in vitro capacitation and HA selection. Furthermore, in accordance with the study by Sakkas et al., in 2003 [11] which showed a correlation between the phosphorylated cell percentage and the fertilisation rate, we could suggest a higher fertilisation success probability using sperm cells from the CS and BS groups.

After assessing the presence of HSPA2 by fluorescence microscopy, we observed that in both US and CS, this protein was immunolocalized in a minority of sperm cells. In contrast, in BS, HSPA2 was present on the surface of almost 70% of sperm cells, while those cells that did not bound to HA displayed HSPA2 immunolabelling in the same proportion as US. Given that spermatozoa are transcriptionally silent and previous studies have shown no loss or gain of HSPA2, our results could indicate that those cells that were able to bind HA have also successfully relocalized HSPA2 on their surface during in vitro capacitation. Similarly, it could be understood that interaction with HA could induce post-translational modifications that may be uncovering HSPA2 epitopes, allowing this protein to be more detectable by fluorescence microscopy in those cells capable of binding to HA, similar to the pattern of C-glycodeline binding [64]. In both cases, the results obtained in this study allows HSPA2 to be proposed as a biomarker of sperm maturity.

In order to describe in depth the HSPA2 immunolocation, we analysed all cells with HSPA2 fluorescence in each experimental group and four different distribution patterns of this protein at cephalic level were observed. Previous reports, however, have characterized a higher number of HSPA2 patterns (i.e. total head, anterior head, posterior head, equatorial band, mid piece, tail, cytoplasm, and membrane) [39]. This discrepancy could be due to different experimental conditions used in both studies since Motiei [39] recorded HSPA2 distribution only over uncapacitated cells and included uncommon patterns. On the contrary, in the present study, only patterns present in more than 5% of cells are taken into account, given that a lower frequency could lead to determine as pattern any artefacts caused by the technique or the heterogeneity of the semen samples [54, 65].

Interestingly, in contrast to US-labelled cells, in which we observed a heterogeneous sperm population regarding HSPA2 location, after incubating under capacitating conditions, we found a more homogeneous population in which the majority of cells showed an equatorial band accompanied by slight fluorescence in the acrosome. Similar location of HSPA2 has been previously illustrated after capacitation by other authors [37, 39, 60, 66] and suggested to have important implications in male fertility.

Once these cells have been exposed to the HA, a significantly higher subpopulation of BS cells presented such distribution in the equatorial band and the acrosomal region. However, the presence of a new distribution pattern in BS group leads us to consider that the contact with HA may be involved in inducing a redistribution in the HSPA2 location to the periacrosomal region of the head. Although this location of HSPA2 has not been the most representative, it is reasonable to associate this distribution with primary gamete recognition, since this event in sperm occurs apically [67].

On the contrary, when we analysed immunofluorescent UBS, we could notice that most of the cells localised HSPA2 in the postacrosome region. This location of HSPA2 has been previously described [39], but the possible involvement in male fertility has not been identified. As the localization of HSPA2 in the postacrosomal region was only observed in US and UBS, this pattern could be a distinctive characteristic of sperm immaturity, such as high frequency of chromosomal abnormalities and cytoplasmic retention, previously described by other authors [60]. Furthermore, the absence of this pattern in the CS group (considered a control for HA selected groups) as well as the presence of an exclusive BS pattern (P4) leads us to reiterate on the suggestion that HSPA2 epitopes might be uncovered through contact with HA.

As a conclusion, according to the results reported in this study, it could be inferred that HA may trigger the uncovering of HSPA2 epitope and induce a redistribution of this protein to the periacrosomal region to play its role in gamete primary recognition [35–37, 68, 69]. These findings, combined with previous reports about HA selecting mature sperm with low levels of chromosomal aneuploidy and DNA fragmentation [18, 21, 24, 25], indicate the suitability of HSPA2 as a biomarker to complement the classic seminal analysis before recommending the appropriate assisted reproductive treatment as long as the use of HA sperm selection prior to artificial reproductive techniques.

However, it would be interesting to further investigate the distribution of HSPA2 in sperm head after different times of capacitation given that, according to our previous studies, a longer capacitation time could lead to post-translational modifications of HSPA2 needed for an optimal capacitation-associated remodeling of the sperm plasma membrane. Moreover, it would be necessary to further investigate the location of the protein complex ARSA/HSPA2/SPAM1 after HA selection of samples from patients performing assisted reproduction techniques, as well as to take into account additional important parameters during fertilization, such as DNA fragmentation [21, 23, 51], membrane integrity [8], or sperm hyperactivation [70].
